# Gut microbiota regulates innate anxiety through neural activity of medial prefrontal cortex in male mice

**DOI:** 10.3389/fnins.2025.1599818

**Published:** 2025-07-24

**Authors:** Jing Ren, Xiao-Ying Lian, Wan-Qian Ye, You-Lu Wen, Cheng-Lin Lu, Xiong Cao

**Affiliations:** ^1^Key Laboratory of Mental Health of the Ministry of Education, Guangdong-Hong Kong-Macao Greater Bay Area Center for Brain Science and Brain-Inspired Intelligence, Guangdong Province Key Laboratory of Psychiatric Disorders, Department of Neurobiology, School of Basic Medical Sciences, Southern Medical University, Guangzhou, China; ^2^Department of Psychology and Behavior, Guangdong Sanjiu Brain Hospital, Institute for Brain Research and Rehabilitation, South China Normal University, Guangzhou, Guangdong, China

**Keywords:** innate anxiety, gut microbiota, neural activity, medial prefrontal cortex, gut-brain-axis

## Abstract

**Introduction:**

Innate anxiety, a stable personality trait conceptualized as trait anxiety, represents a fundamental dimension of individual differences in emotional regulation. Clinical evidence and animal studies indicate that elevated innate anxiety significantly increases susceptibility to psychiatric disorders. While the gut microbiota has been increasingly recognized as a critical modulator of neuropsychiatric health, its specific contribution to innate anxiety has yet to be fully elucidated.

**Methods:**

We investigated gut microbiota contributions to innate anxiety in mice using stratified behavioral phenotyping in the elevated plus maze (EPM), antibiotic (ABX)-mediated microbiota depletion, fecal microbiota transplantation (FMT), c-FOS staining, transcriptomic profiling, and vivo fiber photometry.

**Results:**

We found that innate high-anxiety (HA) and low-anxiety (LA) mice exhibited distinct gut microbial compositions. Microbiota depletion induced significant anxiolytic effects, while FMT from HA donors recapitulated anxiety-like behaviors. Neural activation mapping revealed elevated c-FOS expression in the medial prefrontal cortex (mPFC), basolateral amygdala (BLA), and central amygdala (CeA) of HA-FMT recipients. Transcriptomic analysis of mPFC tissue in HA- and LA-FMT recipients demonstrated microbiota driven regulation of transcriptional reprogramming, protein modification, and synapse modulation, indicating mechanistic connections along the microbiota gut-brain axis. Fiber photometry confirmed heightened mPFC neuronal activity during innate anxiety states in HA-FMT mice.

**Discussion:**

Our findings establish that gut microbiota modulates innate anxiety through mPFC neural activity, providing novel insights into microbiome-based interventions for anxiety.

## 1 Introduction

Innate anxiety is essential for survival in threatening environments. However, pathological anxiety is a prolonged, maladaptive, and exacerbated response to non-threatening situations and greatly affects the quality of life (Price, 2022). In humans, innate anxiety, commonly conceptualized as trait anxiety, constitutes a fundamental and enduring personality dimension that predisposes individuals to demonstrate heightened emotional sensitivity and exhibit persistent patterns of concern and worry in response to various situations. People reporting high trait anxiety have a higher risk of developing psychiatric disorders compared to individuals with low trait anxiety (Chambers et al., 2004; Wang et al., 2019). In mice, innate anxiety is also an instinctive response to avoid potential threats, such as predator exposure, intraspecific conflicts, and adverse environmental stimuli. Innate high anxiety mice intrinsically exhibit elevation in anxiety-related behaviors and provide a valuable experimental model to study anxiety that is not induced by specific stressors (Rooney et al., 2020; Sah et al., 2012). Innate high anxiety have a higher risk of developing anxiety disorders. The etiology of anxiety disorders, long associated with neurochemical factors, brain structure/function, and environmental/genetic/psychological influences, has consequently become a primary focus of scholarly research. Despite extensive research, effective treatment outcomes remain elusive, as current therapies often provide inadequate symptom relief or cause significant adverse effects in many patients. Incomplete understanding of anxiety disorder mechanisms limits treatment efficacy, highlighting the need for mechanistic research to drive therapeutic advances. So, it may also be important for elucidating the mechnism of innate anxiety.

Emerging evidence implicates the gut microbiota as a critical modulator of anxiety pathophysiology (Jiang et al., 2024). Human studies link gut microbiota to mental disorders such as anxiety, with generalized anxiety disorder patients showing distinct microbial profiles (Jiang et al., 2018; Xiong et al., 2023); notably, probiotic supplementation exerts anxiolytic effect (Foster and McVey Neufeld, 2013; Vitellio et al., 2020; El Dib et al., 2021). Probiotics, prebiotics, and FMT can modify the composition of gut microbiota, rebuild the gut environment and improve individuals’ psychological well-being through microbial metabolites (e.g., bioactive compounds for digestion, antimicrobials, and neuroactive molecules) (Antushevich, 2020; Gomaa, 2020; Baske et al., 2024; Noonan et al., 2020; Bear et al., 2020). Preclinical models further establish causal relationships. Germ-free and antibiotic-treated mice exhibit attenuated anxiety-like behaviors (Morais et al., 2021). Fecal microbiota transplantation from anxious BALB/c donors to less anxious NIH Swiss recipients increases both behavioral anxiety and brain-derived neurotrophic factor expression (Bercik et al., 2011). Maternal prebiotic intake in mice reduces anxiety and expression of hippocampal glutamate receptor genes and alters the fecal microbiome in offspring (Hebert et al., 2021). These studies demonstrate that gut microbiota modulates the innate anxiety. Notably, the mPFC, a core element of the neurocircuitry, not only controls fear and anxiety but also plays a role in the microbiota-gut-brain axis to regulate emotional behavior (Jiang et al., 2024; Likhtik et al., 2014; Zhang et al., 2024; Chen et al., 2021; Chu et al., 2019). Nevertheless, the precise mechanisms through which gut microbiota regulate innate anxiety remain elusive.

In this study, innate high-anxiety (HA) and low-anxiety (LA) groups were phenotypically stratified by elevated plus maze (EPM), with classification thresholds established through time spent in the open arms using adult male SPF C57BL/6J mice. We speculated that gut microbiota modulates innate anxiety behaviors via mPFC neuronal activation. This is supported by several lines of evidence in this study. First, distinct gut microbiota compositions differentiate HA and LA phenotypes; Second, microbial depletion via antibiotics exerts anxiolytic effects; Third, HA-FMT recipients display increased anxiety-like behaviors compared to LA-FMT mice; Fourth, elevated c-Fos expression in HA-FMT mice within the mPFC, basolateral amygdala (BLA), and central amygdala (CeA); Fifth, transcriptomic profiling identifies microbiota-dependent regulation of brain function in mPFC; Last, *In vivo* GCaMP recordings demonstrate heightened mPFC neuronal activity during innate anxiety states. Collectively, these findings establish a novel gut-microbiota-mPFC axis governing innate anxiety predisposition.

## 2 Materials and methods

### 2.1 Animals

SPF C57BL/6J mice were housed in groups of three to five per cage under controlled temperature conditions (22°C–25°C) on a 12/12-h light/dark cycle (lights on from 7:00 A.M. to 7:00 P.M.) with *ad libitum* access to food and water. The male C57BL/6J mice (aged 7–8 weeks) were procured from the Southern Medical University Animal Center (Guangzhou, China). Before the commencement of the behavioral experiments, all mice were handled for three consecutive days. The behavioral tests were conducted between 13:00 and 17:00 during the light cycle. All animal protocols were approved by the Southern Medical University Animal Ethics Committee.

### 2.2 Separation of high- and low-anxiety

Mice are stratified into high- and low-anxiety subgroups based on their performance in the EPM test, following established criteria from previous studies (Bi et al., 2015; Ren et al., 2022). The classification protocol consisted of two sequential analytical phases: First, a quantitative behavioral assessment is conducted through standardized EPM testing to measure open-arm exploration time. Subsequently, cohort stratification is implemented using a normalized threshold system - mice demonstrating open arm dwell times exceeding the group mean by ≥ 6% were classified as low-anxiety phenotype, while those exhibiting dwell times ≤ 6% below the mean were classified as high-anxiety phenotype.

### 2.3 ABX treatment

Antibiotics (ABX) treatment was performed as previously described (Olson et al., 2018). In brief, SPF mice were given a solution of vancomycin (50 mg/kg), neomycin (100 mg/kg), and metronidazole (100 mg/kg) by gavage every 12 h daily for 28 days. Ampicillin (1 mg/ml) was provided *ad libitum* in drinking water. For the control group, mice were gavaged with normal drinking water every 12 h daily for 28 days. Behavioral studies were conducted 24 h after the last gavage.

### 2.4 Fecal microbiota transplant

Fecal Microbiota Transplant was performed as previously described (Olson et al., 2018). Fresh fecal samples were collected from high- and low-anxiety mice for fecal microbiota transplantation (FMT) and suspended at 50 mg/ml in pre-reduced PBS. Before FMT, recipient mice were treated with ABX for 7 days to clear gut microbiota. After 3-days washouts, these recipient mice were colonized by oral gavage of 100 ul fecal suspensions from either high- or low-anxiety donor mice. For the control group, ABX-treatment mice were gavaged with pre-reduced PBS. It took 2 weeks to colonize the fecal microbiome before the behavioral tests.

### 2.5 Elevated plus-maze test (EPM)

The EPM apparatus comprised two open arms (30 × 5 × 0.5 cm), two closed arms (30 × 5 × 15 cm), and a central platform (5 × 5 cm) elevated 50 cm above the underlying surface. The experimental protocol entailed the placement of mice in the center of the apparatus, with each mouse facing one of two open arms. The amount of time spent in each arm and arm entries were quantified using EthoVision 11.0 software. This tracking and recording process was conducted for 5 min. Elevated Plus-Maze Test was conducted as described in our previous study (Bi et al., 2015; Ren et al., 2022).

### 2.6 Open field test

The open field test was carried out to evaluate locomotor activity. The apparatus was an opaque square chamber (50 × 50 × 40 cm) with transparent, plastic walls and a white floor. It was divided into two areas: a central field (25 × 25 cm) and an outer field (periphery). Individual mice were placed in the center area of an open chamber at the beginning of the test and allowed to explore freely for 30 min. The total distance and time in the center area traversed by the subjects during a session were subsequently analyzed using Versmax analyzer software. The path taken by each mouse was digitally captured and analyzed using EthoVision 11.0 software. The open field test followed the descriptions in our previous research (Bi et al., 2015; Ren et al., 2022).

### 2.7 Measurement of plasma corticosterone (CORT) levels

Blood samples were drawn by orbital sinus collection and plasma samples were obtained after centrifugation at 3,000 rpm for 3 min. CORT concentrations were measured using a corticosterone enzyme immunoassay kit (Wei Ao Biotechnology Co., Ltd., Shanghai, China) validated for mouse plasma samples according to the manufacturer’s protocol.

### 2.8 16S rRNA gene sequencing

Total microbial genomic DNA was extracted using the E.Z.N.A.^®^ soil DNA Kit (Omega Bio-tek, Norcross, GA, U.S.) according to the manufacturer’s instructions. The hypervariable region V3–V4 of the bacterial 16S rRNA gene was amplified with primer pairs. The PCR product was extracted and purified using the PCR Clean-Up Kit (YuHua, Shanghai, China) according to the manufacturer’s instructions and quantified using Qubit 4.0 (Thermo Fisher Scientific, USA). Purified amplicons were pooled in equimolar amounts and paired-end sequenced on an Illumina Nextseq2000 platform (Illumina, San Diego, USA). Raw FASTQ files were de-multiplexed using an in-house perl script, and then quality-filtered by fastp version 0.19.6 and merged by FLASH version 1.2.7. Then the optimized sequences were clustered into operational taxonomic units (OTUs) using UPARSE 7.1. The most abundant sequence for each OTU was selected as a representative sequence. The OTUs assigned to spike-in sequences were filtered out and reads were counted. The taxonomy of each OTU representative sequence was analyzed by RDP Classifier version 2.2 against the 16S rRNA gene database. The 16S rRNA gene sequencing was conducted by Cosmos Wisdom Co., Ltd., (Hangzhou, China).

### 2.9 RNA sequencing

Total RNA was extracted from the tissue using TRIzol Reagent according to the manufacturer’s instructions (Invitrogen) and genomic DNA was removed using DNase I (Takara). RNA quality was determined by 2100 Bioanalyser (Agilent Technologies) and quantified using the ND-2000 (NanoDrop Technologies). The transcriptome library was prepared following TruSeqTM RNA sample preparation Kit from Illumina (San Diego, CA) using 1 μg of total RNA. Libraries were size-selected for cDNA target fragments of 300 bp on 2% Low Range Ultra Agarose followed by PCR amplified using Phusion DNA polymerase (NEB) for 15 PCR cycles. After quantified by TBS380, the paired-end RNA-seq sequencing library was sequenced with the Illumina NovaSeq 6000 sequencer (2 × 150 bp read length). The raw paired-end reads were trimmed and quality was controlled by fastp with default parameters. Then clean reads were separately aligned to the reference genome with orientation mode using HISAT2 software. The mapped reads of each sample were assembled by StringTie (in a reference-based approach. To identify DEGs (differential expression genes), the expression level of each gene was calculated according to the transcripts per million reads (TPM) method. RSEM was used to quantify gene abundances. DEGs with *P* ≤ 0.05 were considered to be significantly different expressed genes. In addition, functional-enrichment analysis including GO and Reactome was performed to identify which DEGs were significantly enriched in GO terms and metabolic pathways at *P* ≤ 0.05. The RNA sequencing was conducted by Cosmos Wisdom Co., Ltd., (Hangzhou, China).

### 2.10 Fiber photometry

The mice (HA- and LA-FMT) were anesthetized using sodium pentobarbital (50 mg/kg, i.p. injection) for unilateral stereotaxic injection of AAV2/9-Syn-Gcamp6s-WPRE-pA (titer: 2.57 × 10^12^ v.g. ml^–1^; BrainVTA) into the mPFC (AP: + 1.75 mm, ML: + 0.3 mm, and DV: −2.75 mm, 300 nl/site; AP, ML, and DV denote anteroposterior, mediolateral and dorsoventral distances from bregma, respectively). After injection, the needle was left in place for 6 min before being withdrawn at a gradual pace. Following AAV2/9-Syn-Gcamp6s-WPRE-pA virus injection, a ceramic ferrule with an optical fiber [200 μm in diameter, numerical aperture (NA) of 0.37 (Inper)] was implanted with the fiber tip in the mPFC (AP: + 1.75 mm; ML: ± 0.3 mm; DV: −2.5 mm). Signals were recorded 3 weeks after optical fiber implantation.

The fluorescence signals were recorded with a three-color multichannel optical fiber recording system (Thinker Tech, China). The green channel with 470 nm excitation light was used to record the fluorescence of GCaMP6s. To minimize photobleaching, the laser power at the fiber tip was adjusted to 30 μW. For recordings in mice, the fluorescence signals from mice with optical fibers connected to the fiber photometry system were recorded in the EPM. Bulk fluorescence signals were acquired and analyzed with MATLAB software. The z-score was calculated using the following formula: *Z* = (F_Signal_ − F_Basal_)/STD(F_Basal_).

### 2.11 Statistics

All experiments and data analyses were conducted blindly. Statistical analysis were mainly performed using SPSS 20.0 (IBM Corp., USA) with appropriate inferential methods, while GraphPad Prism 9.0 (GraphPad Software, USA) was employed for data visualization, including the generation of statistical graphs. Data are presented as mean ± standard error of the mean (SEM). Significant differences are indicated in the figures by *p < 0.05, **p < 0.01, ***p < 0.001.

## 3 Results

### 3.1 Characterization of high- and low-anxiety subgroups in the EPM

**FIGURE 1:**
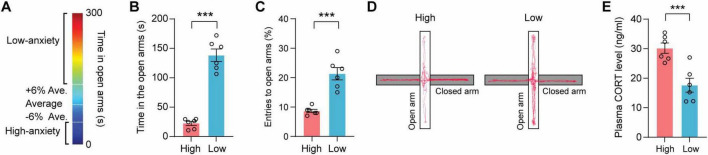
Mice were separated into high- and low-anxiety subgroups in elevated plus maze (EPM). **(A)** Schematic of mice separated into high-anxiety and low-anxiety subgroups. **(B,C)** Time spent in the open arms **(B)** and the ratio of entries to the open arms (**C**) *n* = 6 mice/group. **(D)** Representative tract visualization of mice in EPM. **(E)** The concentration of plasma corticosterone in high and low mice (*n* = 6 mice per group). Data are the mean ± s.e.m. **P < 0.01, ***P < 0.001. *P*-values were determined by a two-tailed unpaired *t*-test.

To investigate the potential involvement of gut microbiota in innate anxiety regulation, specific pathogen-free (SPF) C57BL/6J mice were phenotypically stratified into HA and LA groups using EPM, with classification thresholds established through time spent in the open arms (Figure 1A). It is observed that mice termed LA mice demonstrated prolonged open-arm exploration time compared to HA mice (Figure 1B, *t* = 10.44, *p* = 0.0001). LA mice also showed an increased open-arm entry ratio compared to HA mice (Figure 1C, *t* = 4.133, *p* = 0.0020). Representative tract visualization of HA and LA mice in EPM was illustrated in Figure 1D. Plasma corticosterone levels are elevated in high-anxiety mice Figure 1E. Overall, HA mice displayed significantly enhanced anxiety-like behaviors relative to LA mice.

### 3.2 Gut microbiota composition of high- and low-anxiety mice

**FIGURE 2:**
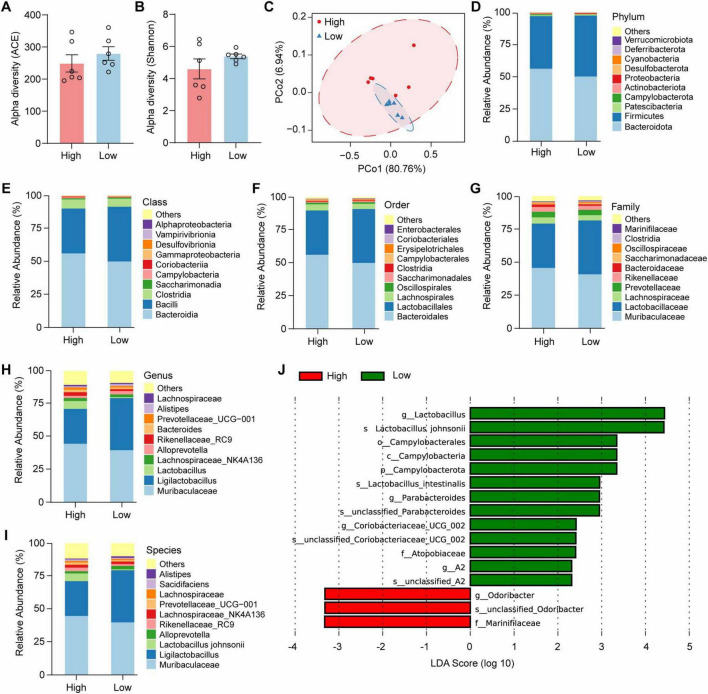
Gut microbiota composition of high- and low-anxiety mice. **(A,B)** Alpha-diversity of the gut microbiota between the high- and low-anxiety mice, as indicated by the ACE **(A)**, and Shannon indices **(B)**. *n* = 6 mice/group. **(C)** Principal coordinates analysis (PCoA) using the Weighted UniFrac distance. *n* = 6 mice/group. **(D–I)** Taxonomic distributions of bacteria at phylum **(D)**, class **(E)**, order **(F)**, family **(G)**, genus **(H)**, and species **(I)** level. **(J)** Analysis of taxonomic abundances using LEfSe. Data are the mean ± s.e.m. *P*-values were determined by a two-tailed unpaired *t*-test.

To examine the composition of the gut microbiota in HA and LA mice, fecal samples of these mice were collected for 16S rRNA gene sequencing (Supplementary Table 1). The composition of the microbiota showed comparable diversity indicated by ACE (Figure 2A, *t* = 0.9014, *p* = 0.3886) and Shannon (Figure 2B, *t* = 1.231, *p* = 0.2466) α-diversity index between HA and LA mice. We applied a principal coordinate analysis (PCoA) of weighted UniFrac distance of beta diversity to reveal global variance patterns in the microbial profiles. PCoA analysis revealed differences in the microbiome composition between HA and LA mice (Figure 2C). At the phylum level, we detected *Bacteroidota, Firmicutes, Verrucomicrobiota, Deferribacterota, Cyanobacteria, Desulfobacterota, Proteobacteria, Actinobacteriota, Campylobacterota, Patescibacteria*, and others (Figure 2D). The relative abundance of the microbiota was also shown at class (Figure 2E), order (Figure 2F), family (Figure 2G), genus (Figure 2H), and species (Figure 2I) levels. Notably, several strains showed different abundances between HA and LA mice (Figure 2J). The relative abundance of *Lactobacillus*, *Campylobacterota*, and *Parabacteroides* was increased in the LA mice (Figure 2J). Together, these results suggested that gut microbiota showed a different composition between HA and LA mice.

### 3.3 ABX treatment produced anxiolytic effects in mice

**FIGURE 3:**
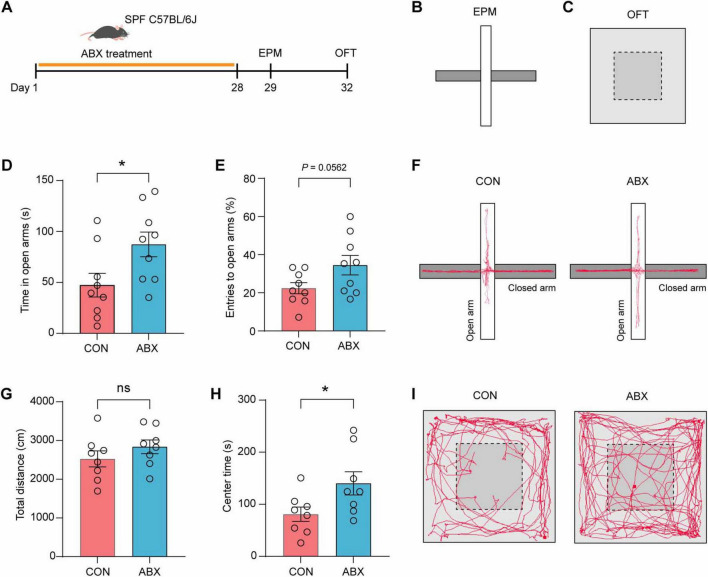
ABX treatment produced anxiolytic effects in mice. **(A–C)** Schematic of mice treated with ABX. SPF C57BL/6J mice were orally treated with ABX for 28 days and then subjected to test anxiety-related behaviors in EPM **(B)** and OFT **(C)**. **(D,E)** Time spent in the open arms **(D)** and the ratio of entries to the open arms **(E)** of mice treated with vehicle and ABX in EPM. *n* = 9 mice/group. **(F)** Representative tract visualization of mice treated with vehicle and ABX in EPM. **(G,H)** Total distance and center time of mice treated with vehicle and ABX in OFT. *n* = 8 mice/group. **(I)** Representative tract visualization of mice treated with vehicle and ABX in OFT. Data are the mean ± s.e.m. ns, no significant, *P < 0.05. *P*-values were determined by a two-tailed unpaired *t*-test.

To determine the effect of gut microbiota on anxiety-like behaviors, SPF mice underwent a 28-days antibiotic (ABX) treatment via oral gavage (Figure 3A). Subsequently, anxiety-related behaviors were evaluated through a standardized behavioral test battery (Figures 3B, C). We found that ABX-treated mice demonstrated significantly prolonged open-arm exploration time in EPM (Figure 3D, *t* = 2.382, *p* = 0.03) and a marginally increased open-arm entry ratio compared to vehicle controls (Figure 3E, *t* = 2.058, *p* = 0.0562). Representative tract visualization of mice treated with vehicle and ABX in EPM was illustrated in Figure 3F. Open field test (OFT) analyses showed comparable total locomotion distances between groups (Figure 3G, *t* = 1.157, *p* = 0.2667), but ABX-treated mice exhibited significantly enhanced center zone exploration time (Figure 3H, *t* = 2.263, *p* = 0.0401). Representative tract visualization of mice treated with vehicle and ABX in OFT was illustrated in Figure 3I. Together, these results suggested that ABX treatment produced anxiolytic effects in mice.

### 3.4 Fecal transplanted microbiome affects anxiety-related behaviors

**FIGURE 4:**
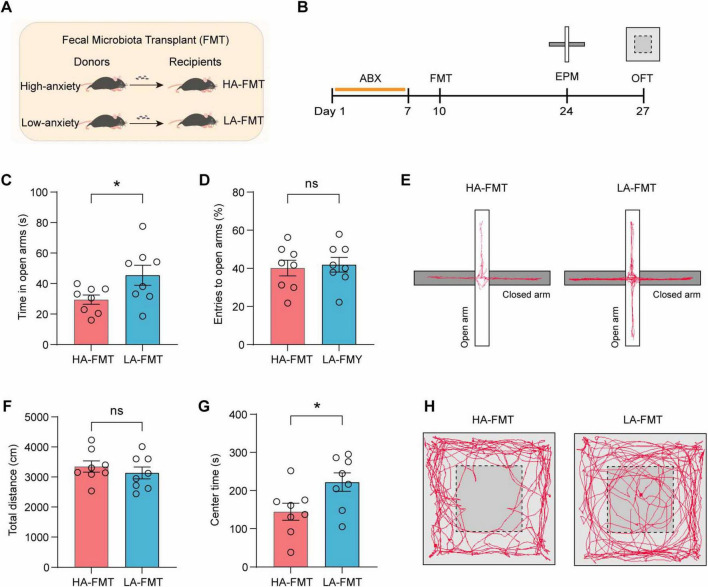
Anxiety-related behaviors of mice transplanted with fecal microbiome from high- and low-anxiety mice. **(A)** Schematic of mice transplanted with fecal microbiome from high- and low-anxiety mice. **(B)** Experimental design. **(C,D)** Time spent in the open arms **(C)** and the ratio of entries to the open arms **(D)** of mice transplanted with fecal microbiome from high- and low-anxiety mice in EPM. *n* = 8 mice/group. **(E)** Representative tract visualization of mice in EPM. **(F,G)** Total distance **(F)** and center time **(G)** of mice transplanted with fecal microbiome from high- and low-anxiety mice in OFT. *n* = 8 mice/group. **(H)** Representative tract visualization of mice in OFT. Data are the mean ± s.e.m. ns, no significant, *P < 0.05. *P*-values were determined by a two-tailed unpaired *t*-test.

To establish causality between gut microbiota composition and anxiety phenotypes, ABX-treated mice received fecal microbiota transplantation (FMT) from donor mice exhibiting high-anxiety or low-anxiety behavioral profiles (Figure 4A) and then subjected to behavioral studies to assess anxiety-related behaviors (Figure 4B). HA-FMT recipients showed reduced open arm exploration time in EPM compared to LA-FMT recipients (Figure 4C, *t* = 2.204, *p* = 0.0448), though open arm entry ratios remained comparable (Figure 4D, *t* = 0.3124, *p* = 0.7593). Representative tract visualization of mice in EPM was illustrated in Figure 4E. In the OFT, while the total distance traveled showed no significant differences between groups (Figure 4F, *t* = 0.7933, *p* = 0.4409), HA-FMT recipients exhibited a significant reduction in center zone exploration time compared to LA-FMT recipients (Figure 4G, *t* = 2.366, *p* = 0.0329). Representative tract visualization in OFT was illustrated in Figure 4H. Together, these findings suggested that the gut microbiota modulated anxiety-like behaviors.

### 3.5 c-FOS^+^ expression in anxiety-candidate brain regions

**FIGURE 5:**
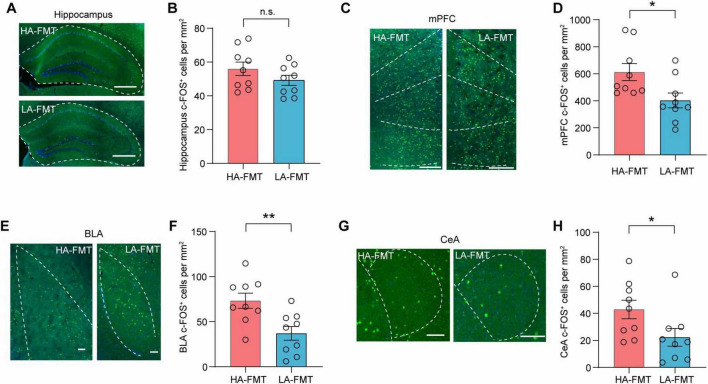
c-FOS^+^ expression in anxiety candidate brain region of mice transplanted with fecal microbiome from high- and low-anxiety mice. **(A,B)** Represented images **(A)** and quantification **(B)** of c-FOS^+^ cells in the hippocampus. Scale bars, 500 μm. *n* = 9 slices from 3 mice. **(C,D)** Represented imagines **(C)** and quantification **(D)** of c-FOS^+^ cells in the mPFC. Scale bars, 500 μm. *n* = 9 slices from 3 mice. **(E,F)** Represented imagines **(E)** and quantification **(F)** of c-FOS^+^ cells in the BLA. Scale bars, 100 μm. *n* = 9 slices from 3 mice. **(G,H)** Represented imagines **(G)** and quantification **(H)** of c-FOS^+^ cells in the CeA. Scale bars, 500 μm. *n* = 9 slices from 3 mice. Data are the mean ± s.e.m. ns, no significant, *P < 0.05 and **P < 0.01. *P*-values were determined by a two-tailed unpaired *t*-test.

To elucidate the neurofunctional consequences of anxiety-associated gut microbiota, we conducted a comparative c-FOS immunohistochemical analysis of anxiety-candidate brain regions in recipient mice receiving FMT from HA versus LA donors after 1 h of EPM test. Based on established correlates of anxiety with the hippocampus, mPFC, BLA, and CeA, we systematically quantified neuronal activation patterns. We found that the c-FOS expression in the hippocampus was not significantly changed between HA-FMT and LA-FMT mice (Figures 5A, B, *t* = 1.362, *p* = 0.1920). In the mPFC, c-FOS^+^ cells of LA-FMT mice were decreased compared to HA-FMT mice (Figures 5C, D, *t* = 0.2519, *p* = 0.0228). LA-FMT also exhibited a reduction in c-FOS^+^ cells in the BLA (Figures 5E, F, *t* = 3.184, *p* = 0.0058) and CeA (Figures 5G, H, *t* = 2.161, *p* = 0.0462). These results showed that anxiety-associated gut microbiota affect brain function in mice.

### 3.6 Transcriptomic analysis of the mPFC from HA- and LA-FMT mice

**FIGURE 6:**
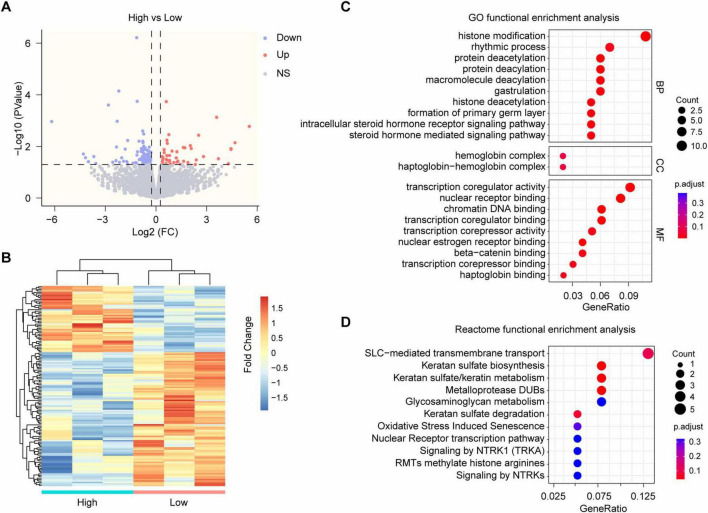
RNA-sequence analysis of mPFC in mice receiving fecal microbiota transplants from high- and low-anxiety donors. **(A)** Volcano plot of the differentially expressed genes (DEGs) of mPFC in mice transplanted with fecal microbiome from high- and low-anxiety mice. *n* = 3 mice/group. **(B)** Hierarchical clustering based on the expression profiles of DEGs. **(C)** GO functional enrichment analysis of DEGs. **(D)** Reactome functional enrichment analysis revealed a significant overlap of DEGs.

Under physiological conditions, mPFC inhibits top-down regulation of amygdala activity. Under adverse circumstances such as chronic stress, the control ability of mPFC is decreased, which leads to abnormal activation of the amygdala and emotional and behavioral disorders (Chen et al., 2021; Liu et al., 2020; Gao et al., 2023). Therefore, we conducted transcriptomic profiling of the mPFC in FMT recipients from HA-FMT versus LA-FMT donors to delineate the molecular mechanisms underlying gut microbiota-mediated neural modulation. RNA sequencing revealed 139 differentially expressed genes between cohorts, comprising 46 upregulated and 93 downregulated transcripts in HA-FMT recipients relative to LA-FMT controls (Figures 6A, B) (Supplementary Table 2). Then, gene ontology (GO) functional enrichment analysis of the DEGs was performed, which revealed enrichment in each of the biological processes (BP), cellular components (CC), and molecular functions (MF) (Figure 6C). The enriched BP was mainly relevant to histone modification, protein acetylation, and steroid hormone receptor signaling pathways. The enriched CC was hemoglobin complex and haptoglobin−hemoglobin complex. The enriched MF was mainly related to the transcription coregulator activity, nuclear receptor binding, and nuclear estrogen receptor binding (Figure 6C). Furthermore, reactome functional enrichment analysis revealed a significant overlap of DEGs with 5 pathways (Figure 6D). The most significant enrichment was solute carrier (SLC)−mediated transmembrane transport (Figure 6D). The convergence of regulation of transcription, protein acetylation, steroid signaling, and transmembrane transport pathways suggested a gut microbiota-brain axis mechanism involving transcriptional reprogramming, modification of proteins, and synapse modulation.

### 3.7 Gut microbiota affects neuronal activity in mice

**FIGURE 7:**
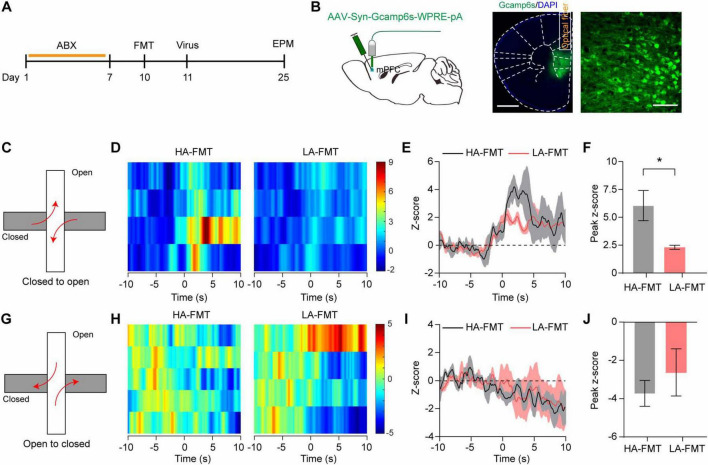
Medial prefrontal cortex (mPFC) neuron activity in mice receiving FMT from high- and low-anxiety donors. **(A)** Experimental design. **(B)** Schematic of fiber photometry and represented images of Gcamp6s expression and optical fiber above the mPFC in mice transplanted with fecal microbiome from high- and low-anxiety mice. Scale bar, 1000 μm (left) and 100 μm (right). **(C)** Schematic of mice transition from closed to open arms. **(D)** Example heatmaps of normalized Ca^2+^ activity in mPFC neurons before and after mice transition from closed to open arms (10 s interval). **(E,F)** A line graph of average changes **(E)** and statistics of maximum changes **(F)** in normalized Ca^2+^ activity. *n* = 4 mice/group. **(G)** Schematic of mice transition from open to closed arms. **(H)** Example heatmaps of normalized Ca^2+^ activity before and after 10 s of mice moved from open to closed arms. **(I,J)** A line graph of average changes **(I)** and statistics of maximum changes **(J)** in normalized Ca^2+^ activity. *n* = 4 mice/group. Data are the mean ± s.e.m. ns, no significant, *P < 0.05. *P*-values were determined by a two-tailed unpaired *t*-test.

Given that the transcriptomic analysis of the mPFC from mice transplanted with anxiety-associated microbiome, gut microbiota may affect neuronal function in the mPFC. Therefore, we next employed *in vivo* fiber photometry to quantify real-time neuronal dynamics in the mPFC of FMT recipients in EPM (Figure 7A). We injected the AAV-Syn-GCaMP6s virus into the mPFC and then implanted optical fibers above the mPFC for recordings of GCaMP fluorescence (Figure 7B). To evaluate the Ca^2+^ activity of neurons during the exploration of innately anxiogenic environments in EPM, fluorescence signals from mPFC neurons were measured when mice moved between the arms (Figure 7C). Heatmaps showed that the activity of mPFC neurons significantly increased when mice transitioned from the closed arms to the open arms in HA-FMT recipients (Figure 7D). The average and maximum GCaMP fluorescence changes (Z-score) were significantly higher in HA-FMT recipients than in LA-FMT recipients when mice moved from the closed arms to the open arms, indicating an innate anxious state (Figures 7E, F, *t* = 2.74, *p* = 0.0337). Although mPFC neuron activity decreased marginally in HA-FMT compared to LA-FMT recipients during the open-to-closed arm transition, the change was not statistically significant (Figures 7G–J, *t* = 0.7787, *p* = 0.4657). These findings demonstrated that gut microbiota affected mPFC neuron activity.

## 4 Discussion

Growing research highlights the pivotal role of microbiota in modulating brain function and behavioral responses. The results of this study provide evidence for a microbiota-gut-brain axis that influences mPFC neural activity and modulates innate anxiety behavior in adult male C57 mice. Some strains of mice displayed enhanced innate anxiety in comparison with C57BL/6 mice (Ugursu et al., 2024). We identified innate high- or low-anxiety phenotypes of C57BL/6 mice in the EPM. Our data indicate that the relative abundance of *Lactobacillus, Campylobacteria, and Parabacteroides* was decreased in the high-anxiety mice. Consistent with the clinical trial and mice model, studies indicated probiotic *Lactobacillus* could relieve anxiety (Lew et al., 2019; Zhu et al., 2023; Weng et al., 2024). In line with previous reports (Foster and McVey Neufeld, 2013; Cryan et al., 2019), we also found ABX-treated mice showed reduced anxiety-like behavior. The importance of considering gender and age in research exploring the role of gut microbiota in mood disorders has been recognized (Clarke et al., 2012; Desbonnet et al., 2015; Leclercq et al., 2017; Bayrer et al., 2023; Bridgewater et al., 2017; Zhang et al., 2021). This investigation did not specifically examine the gut microbiota composition of female mice and developmental phases (neonatal and juvenile), highlighting the necessity for subsequent research to investigate the impact of age and sex on the microbiome in innate anxiety behavior. While the limitation in the paper was that FMT studies were not conducted in germ-free (GF) mice primarily due to cost-effectiveness, as GF models demand specialized isolators and higher resources. Conventional SPF mice provide a practical model with a fully developed immune system, enabling investigation of microbiota-immune dynamics in a physiologically relevant context (Ganal et al., 2012). However, the inherent limitation lies in the resident microbiota potentially impeding donor engraftment and complicating result interpretation through competitive interactions; this was mitigated by standardized antibiotic pretreatment to optimize colonization. From a scientific perspective, complementary GF mouse studies are valuable for assessing the direct functional impact of transplanted microbiota without confounding pre-existing commensals.

The mPFC is widely accepted as a key component of the neural circuitry underlying anxiety-related behaviors. In human post-traumatic stress disorder and generalized anxiety disorder, the mPFC is a critical site for anxiety (Greenberg et al., 2013; Rauch et al., 2006). In primates, anxiety can be regulated by the prefrontal cortex (PFC) (Kenwood et al., 2022). In rodents, mPFC-involved circuits including BLA (Liu et al., 2023; Felix-Ortiz et al., 2016; Shen et al., 2023; Shen et al., 2019), CeA (Chen et al., 2021), and hippocampus (Adhikari et al., 2011; Adhikari et al., 2010) encode stress and anxiety. Subdiaphragmatic vagotomy (Klarer et al., 2014) and chronic vagal afferent lesions (Krieger et al., 2022) both impair gut-to-brain signaling, reducing anxiety-like behaviors via GABAergic dysregulation in the PFC and amygdala, respectively. These findings highlight the critical role of gut and some brain regions in anxiety regulation. In accordance with above, we not only observed an increased cFOS expression in the mPFC, BLA, and CeA of the high-anxiety FMT recipients with anxiety-like behaviors but also noted a significant abundance of cFOS expression in the mPFC. Combining with transcriptomic analysis that gut microbiota may affect neuronal function in the mPFC, we utilized *in vivo* fiber photometry to quantify real-time neuronal dynamics and observed an increase in neural activity within the mPFC of HA-FMT recipients when mice transitioned from closed to open arms in the EPM, indicating an innate anxious state. Whereas, this study did not form a connection between the brain and vagus nerve in the modulation of anxiety, especially gut microbiota as major sources of stimulation. Thus, it is necessary for us to map out the up-downstream circuits of gut-bain and explore the precise molecular and circuitry mechanisms underlying the regulation of innate anxiety in future studies.

Gut microbiota may contribute to anxiety regulation through multiple pathway-microbial metabolism including neurotransmitters (metabolized by gut microbiota), endocrine pathways, immune responses (inflammatory metabolites produced by gut microbiota) and so on. To elucidate the gut microbiota-brain axis mechanism, we conducted RNA sequencing of mPFC in mice transplanted with fecal microbiome from high and low-anxiety mice and revealed the involvement of SLC transporters. Nonetheless, we may continue to explore the mechanisms through which microbiota-derived metabolites regulate cellular activity and innate anxiety in the mPFC. Previous research has shown that microbiota-derived metabolites impact microglia-mediated synaptic pruning, thereby indirectly affecting excitatory neurons within the medial prefrontal cortex and modulating fear extinction behavior (Chu et al., 2019). Furthermore, microglia serve as a potential target for addressing and resolving microglia heterogeneity in mice exhibiting innate high anxiety (Ugursu et al., 2024). Additionally, gut microbial short-chain fatty acids (SCFAs) and polyunsaturated fatty acids (PUFAs) play a role in modulating the innate anxiety response (Ren et al., 2022; Wu et al., 2022). Therefore, microbiota-derived metabolites (i.e., fatty acids) may also exert an influence on other cell subsets in the mPFC (such as microglia), thereby indirectly modulating excitatory neurons and innate anxiety behavior. Morever, the the specific mechanism is complex. Jiang et al. (2024) eluciate the role and recent advancements in the microbiota-gut-brain axis concerning the mechanism of anxiety disorders through neurotransmitters (e.g., GABA, DA, NE, and 5-HT etc., metabolized by gut microbiota), endocrine pathways [e.g., glucocorticoids (GCs), short-chain fatty acids (SCFAs), fatty acid amides (FAAs), 4-ethylphenylsulfate (4EPS), and secondary bile acids (SBAs) etc.], and immune responses (pro-inflammatory and anti-inflammatory metabolites produced by gut microbiota). New gut microbiota metabolites related to anxiety may continue to be discovered, offering new insights into the pathophysiological mechanisms of anxiety and the development of novel therapeutic strategies.

In summary, this study establishes a gut-microbiota-mPFC neural axis as a key regulator of innate anxiety. We demonstrate that gut microbial composition modulates anxiety behaviors through mPFC neuronal activity, with distinct microbial signatures differentiating high- and low-anxiety phenotypes. Our findings reveal that microbial depletion alleviates anxiety while fecal microbiota transplantation transfers anxiety traits, accompanied by microbiota-dependent mPFC hyperactivity. Taken together, our findings demonstrate that gut microbiota regulates anxiety by modulating brain neuronal activity, highlighting microbiota-targeted therapies could restore gut-brain communication and alleviate anxiety-related disorders.

## Data Availability

The raw data of 16S rRNA gene sequences has been deposited to NCBI Sequence Read Archive under BioProject PRJNA1229838. Data of RNA-seq has been deposited in the Gene Expression Omnibus under Accession Number GSE290997.
